# High Dose Intravenous Vitamin C as Adjunctive Therapy for COVID-19 Patients with Cancer: Two Cases

**DOI:** 10.3390/life12030335

**Published:** 2022-02-24

**Authors:** Guangling Guo, Qi Chen, Guoshi Luo, Zhongji Meng, Pan Lei, Ping Chen, Jeanne A. Drisko

**Affiliations:** 1 Anti-Aging Medical Center, Taihe Hospital, Hubei University of Medicine, Shiyan 442000, China; 2 Department of Pharamcology, Toxicology and Therapeutics, School of Medicine, University of Kansas Medical Center, Kansas City, KS 66160, USA; qchen@kumc.edu (Q.C.); pchen2@kumc.edu (P.C.); jdrisko@kumc.edu (J.A.D.); 3 Department of Pulmonary and Critical Care Medicine, Taihe Hospital, Hubei University of Medicine, Shiyan 442000, China; guoshi.luo@taihehospital.com; 4 Department of Infectious Diseases, Taihe Hospital, Hubei University of Medicine, Shiyan 442000, China; zhongji.meng@taihehospital.com; 5 Hubei Key Laboratory of Wudang Local Chinese Medicine Research, Hubei University of Medicine, Shiyan 442000, China; pan.lei@taihehospital.com

**Keywords:** COVID-19, intravenous vitamin C, IVC, cancer, infection

## Abstract

Background: Related to the SARS-CoV-2 pandemic leading to COVID-19 illness, patients with cancer comorbidity are known to have a higher risk of developing severe viral-related events, including death. To date, there are few treatments with proven efficacy for COVID-19. Vitamin C administered intravenously (IVC) has been extensively investigated in cancer treatment with a known safety profile and has been proposed to play a role in managing COVID-19. IVC was used to treat COVID-19 patients in hospitals in China, USA, and Europe with reported benefits. We report here unexpected beneficial results from the use of IVC in two severely ill oncology patients with documented COVID-19 lung disease. Case Report: two oncology patients were diagnosed with SARS-CoV-2 infection. Prior to receiving IVC, lung infiltrates and systemic inflammation in both patients were progressing despite multiple anti-viral, antibiotic, and anti-inflammatory treatments with intensive supportive care. Both patients subsequently received 12 g of IVC delivered intravenously over 30 min, given 2 times daily for 7 days. Serial SARS-CoV-2 nucleic acid tests showed that the viral load was negative only after the 7-day IVC treatment. In both patients after receiving IVC infusions, imaging by chest CT or X-ray showed improving lung infiltrates. There were reductions in systematic inflammation by high-sensitivity C-reactive protein (hsCRP), and Interleukin-6 (IL-6) testing. No adverse events were observed related to IVC treatment. Conclusion: the use of high-dose IVC demonstrated unexpected clinical benefits in treating COVID-19 in two cancer patients presenting with complicated severe comorbidities where an unfavorable prognosis was anticipated.

## 1. Introduction

In December 2019, an outbreak of a coronavirus viral infection, SARS-CoV-2, led to a coronavirus infectious disease (COVID-19) that spread rapidly around the world. On 11 March 2020, the World Health Organization (WHO) declared COVID-19 outbreak a pandemic [1]. Globally, numbers of confirmed cases have reached over 373 million as of January 2022 with a death toll over 5.6 million [1]. Currently, new cases of COVID-19 are being diagnosed daily. Although vaccines have been developed and deployed, variants of the virus have been detected, leading to ongoing waves of the pandemic. COVID-19 patients may develop severe diseases that have a high fatality rate, especially in the elder and those with comorbidities [2,3]. 

Patients with cancer are usually an older age, have multiple complicated comorbidities with compromised immunity, and are at high risk for developing SARS-CoV-2 infection. This risk includes developing severe symptoms with increased mortality once infected, especially for those currently receiving or recently undergoing cytoreductive therapies. A study supported the increased risk for cancer patients who had chemotherapy or surgery within a month of COVID-19 diagnosis having a numerically higher risk of clinically severe events such as ICU admission, invasive ventilation, or death compared to COVID-19 patients without a cancer diagnosis [4]. The median time to progression to COVID-19 related severe clinical events was 13 days in cancer patients compared to 43 days in patients without cancer [4]. 

The overwhelming urgency of COVID-19 demands finding effective methods for controlling the spread and to treat the infected. Potential treatments for COVID-19 are under clinical investigation or have been approved, including vaccines, antibodies, anti-viral medicines, experimental compounds, and plasma from recovered COVID-19 patients [5]. More than one strategy may prove its worth, and effective treatments may work at different stages of the infection [5]. Supportive therapy remains the main method of treatment in addition to general anti-viral and anti-bacterial therapies. 

Ascorbic acid (vitamin C) has been proposed as a potential treatment modality for COVID-19 patients, based on its historical uses and recent reports in treating infection [6,7,8,9]. High-dose intravenous vitamin C (IVC) has an extraordinary safety profile [10,11,12], and, in translational oncology, research has shown clinical benefits [13,14]. IVC in controlled clinical trials at doses of 75–100 g/infusion and infused at a rate of 0.5 g/min was shown to be safe and well-tolerated by cancer patients [10,11,12]. Several clinical trials using IVC in COVID-19 and other infections are listed at ClinicalTrials.gov website: (https://www.clinicaltrials.gov/ct2/results?cond=Infections+&term=intravenous+vitamin+C&cntry=&state=&city=&dist= (accessed on 2 February 2022). Furthermore, IVC has been used by hospitals in China, USA, and European countries in COVID-19 treatment, and beneficial results have been reported [15,16,17]. 

Herein we report two cancer patients with severe COVID-19 treated at Taihe Hospital, Shiyan City, Hubei Provence, China using IVC as adjunctive therapy in addition to supportive care and general anti-infective therapies. Neither patient was expected to survive. In these two cases, both patients had an unexpected recovery after IVC treatment.

## 2. Case Report

### 2.1. Case 1

**Figure 1:**
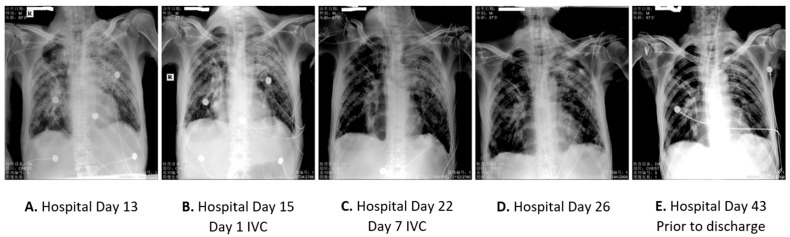
Chest X-ray images of patient 1. Hospital days 15–22 patient received IVC infusions. (**A**) Hospital Day 13 with worsening status and intubation. (**B**) Hospital Day 15 = Day 1 on IVC. (**C**) Hospital Day 22 = Day 7 on IVC. (**D**) Hospital Day 26 discharge from COVID-19 Unit. (**E**) Follow-up X-ray prior to hospital discharge.

A 64-year-old male presented to urgent care in February 2020 with several days of cough and wheeze, difficulty expectorating sputum, mild dyspnea, and chest pain; he was subsequently hospitalized. The patient was cachectic at admission and had mild dyspnea with a respiratory rate (RR) of 20–25/min. He had a history of esophageal cancer with radical esophagectomy performed in October 2019. The patient had an over 50-pack year history of tobacco use and daily alcohol intake (100 mL/day) but reported discontinuing both after surgery. He was hyperglycemic (fasting blood glucose 6.5–14.1 mmol/L) at admission, although he denied diabetes history. Initial plain film chest X-ray described postoperative anastomotic-pleural fistula, right pleural effusion, and bilateral lung infiltrates. Oropharyngeal swab tests for SARS-CoV-2 nucleic acids were positive on three separate days and positive for influenza B virus. The arterial blood gases (ABGs), complete blood count, and basic metabolic profiles were monitored. The patient had lymphopenia and anemia with elevated neutrophil counts. High-sensitivity C-reactive protein (hsCRP) was elevated to 198 mg/L, and serum amyloid A was elevated to 512 mg/L (Table 1), indicating acute inflammation. The patient received anti-viral treatments of α-interferon and arbidol hydrochloride (0.2 g p.o. q8h), antibiotics, heparin (12,500 U), nutritional support, and symptomatic supportive care. After 7-days of treatment, Influenza B virus was negative, but SARS-CoV-2 remained positive. The lung infiltrates and dyspnea progressed (RR 19–36/min). SPO_2_ was 90% on nasal cannula with O_2_ at 5 L/min. On Hospital Day 8, the patient had worsening dyspnea with a respiratory rate of 43 BPM and a pulse rate of 114 BPM. High flow oxygen through a face mask (40 L/min) was needed to maintain SPO_2_ > 90%. The patient was found to have superimposed pseudomonas aeruginosa and staphylococcus aureus pulmonary infections. Laboratory analysis showed worsening lymphopenia, inflammatory indicators, and metabolic profile (Table 1). In the following week, his conditions deteriorated despite acetylcysteine lung lavage, serum therapy from a COVID-19 survivor, continued α-interferon, arbidol hydrochloride, multiple antibiotics, blood transfusions, and other intensive care support for symptoms. On Hospital Day 13, the patient progressed to acute respiratory failure and loss of consciousness with SPO_2_ at 74%, and PCO_2_ elevated to 120.7 mmHg. The patient was intubated and placed on mechanical ventilator support to maintain SPO_2_ > 95%; respiratory acidosis and metabolic alkalosis developed. The patient was moribund with signs of heart failure (brain natriuretic peptide (BNP) 1100–2000 pg/mL) and renal failure (Table 1). Pupils dilated to 3 mm, and light reflex was weak. Chest X-ray imaging showed significant progression of lung infiltrates with increased patchy densities and honeycomb-like changes (Figure 1A). The patient’s SARS-CoV-2 test remained positive.

**Figure 2:**
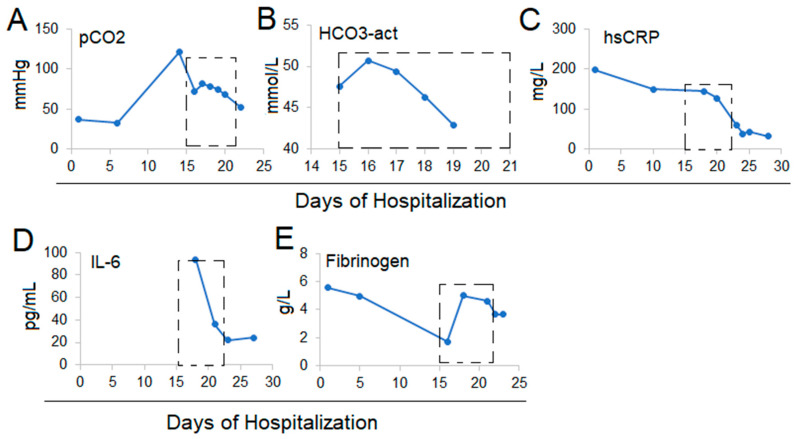
Patient 1. Laboratory changes pCO_2_ (**A**), HCO_3_ (**B**), hsCRP (**C**), IL6 (**D**), and Fibrinogen (**E**). The boxes with dotted lines represent Hospital Days 15–22 on intravenous vitamin C.

On Hospital Day 15, the chest X-ray showed ongoing lung infiltrates (Figure 1B) as the patient was rapidly declining. Intervention with intravenous vitamin C (IVC) was begun at a dose of 12 g infused over 30 min and given every 12 h for 14 infusions over 7 days. The anti-viral, anti-bacterial, and supportive treatments were continued. After 24 h of the IVC treatment, pCO2 decreased and continued to decrease along with blood HCO3-act during the 7-day IVC treatment (Figure 2A,B) (Table 1). Gas exchange gradually improved. The hsCRP decreased from the initial value of 149 mg/L to 61 mg/L at day 7 of the IVC treatment (Figure 2C) (Table 1). The initial elevated neutrophil counts (8.27, 93.7%) normalized (5.81, 89.3%) (Table 1). IL-6 decreased from 94 to 21.95 (Figure 2D) (Table 1). Fibrinogen normalized (Figure 2E) (Table 1). Chest X-ray showed improvement in the bilateral lung infiltrates (Figure 1C). There was no change in D-dimer protein, BNP, and prothrombin time. SARS-CoV-2 tests were negative at the end of the IVC treatment. A follow-up chest X-ray 4 days after the completion of IVC treatment on Hospitalization Day 26 showed improved bilateral interstitial lung disease (ILD) and resolving lung infiltrates (Figure 1D). The patient’s clinical condition improved and stabilized. The patient was released from the COVID-19 unit on Hospital Day 27 and transferred to a pulmonary specialty department, where supportive care continued. A follow-up chest X-ray prior to discharge showed ongoing improvement of lung disease (Figure 1E), and the patient was released from the hospital shortly thereafter.

### 2.2. Case 2

**Figure 3:**
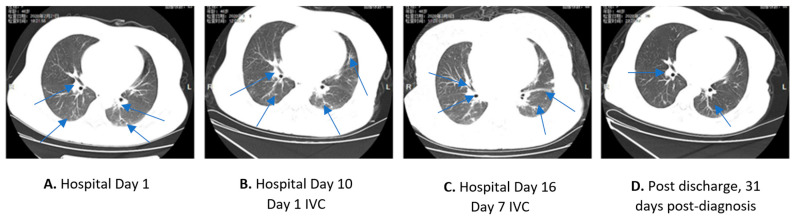
Patient 2. Unenhanced high-resolution CT (HRCT) of the chest during hospitalization (**A**–**C**). Post-discharge HRCT scan 31 days post-diagnosis (**D**). Arrows In (**A**–**D**) show the patchy infiltrates and consolidation. Patchy infiltrates remain at the termination IVC and likely represent residual inflammation and cellular debris (**C**). Patient was discharged from the COVID-19 ICU on Hospital Day 18. The lung findings largely cleared 31 days post-diagnosis but with some residual infiltrates (**D**).

A 46-year-old female patient presented to the outpatient department of the hospital in February 2020 with dizziness, weakness, and chest tightness. She gave a history of a visit in the prior month from her son, who traveled from Wuhan. At the time of the visit, the patient was undergoing chemotherapy (oral Imatinib 600 mg daily) for chronic myelogenous leukemia (CML). She also had comorbidities of diabetes and anemia. At presentation, the patient did not have fever, cough, myalgias, diarrhea, or dyspnea. Initial unenhanced high-resolution CT (HRCT) of the chest demonstrated bilateral lung infiltrates (Figure 3A). Laboratory measurements of the arterial blood gas (ABGs), complete blood count (CBC), and basic metabolic profile are shown in Table 2. Oropharyngeal swab tests were positive for both SARS-CoV-2 and Influenza B virus. The patient had lymphopenia and anemia. The hsCRP was elevated to 36.25 mg/L (Table 2). The patient was hospitalized and treated with anti-viral oseltamivir 75 mg po q2, antibiotics—moxifloxacin IV, and glycyrrhizinate 150 mg po q3 (anti-inflammation). Imatinib was continued. The patient also received oral vitamin (C 500 mg po q3) and Traditional Chinese Medicine (TCM) Lianhuang Qingwen capsules (2 capsules po q3). Her presenting symptoms worsened, and she was admitted to the COVID-19 ICU on Hospital Day 3. Anti-viral treatment changed to arbidol hydrochloride (0.2 g po q3) and ribavirin (0.5 g IV q2), and antibiotics changed to IV piperacillin and tazobactam. The glycyrrhizinate, TCM, and oral vitamin C continued at the same doses. During the hospitalization, the patient received several blood transfusions to treat the anemia. COVID-19 survivor serum (150 mL) was given on Hospital Day 7. 

The patient’s condition deteriorated (Table 2), and she developed dyspnea, myalgias, diarrhea, and nausea and vomiting. On Hospital Day 9, laboratory testing of Influenza B virus and bacteria were negative, but SARS-CoV-2 remained positive. The patient’s total white blood cell count (WBC) elevated to 18.09 g/L, neutrophil count elevated to 14.87 g/L, and lymphopenia continued (1.09, 6.4%). The oral imatinib was discontinued, and the possible recurrence of leukemia was questioned with the elevated WBC. The hsCRP increased to 63 mg/L and procalcitonin was also elevated (0.54 mg/L). The patient required nasal oxygen via cannula to maintain SPO_2_ > 95%. The lung infiltrates progressed on an unenhanced HRCT scan with thickened bilateral bronchial vascular bundles, and multiple patchy high-density infiltrates throughout (Figure 3B). 

**Figure 4:**
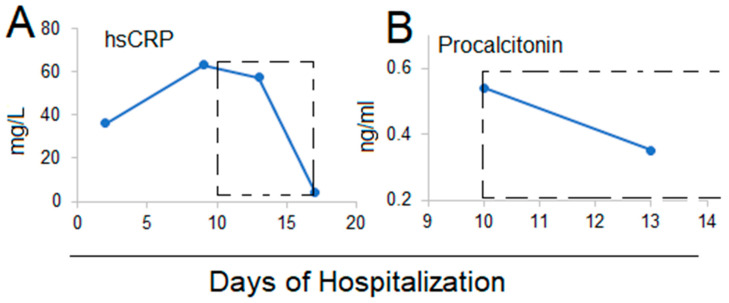
Changes of hsCRP (**A**) and procalcitonin (**B**) in patient 2. The boxes with dotted lines show the days when patients received the IVC infusions (Day 10–16 of hospitalization).

On Hospital Day 10, IVC was begun at a dose of 12 g infused over 30 min, given every 12 h for 7 days, in addition to ongoing intensive supportive care. Within 48 h of beginning the IVC treatment, nausea, vomiting, and diarrhea improved. On Hospital Day 13, 3 days after beginning IVC treatment, the SARS-CoV-2 test was negative and remained negative on the following days. After the 7-day IVC treatment, an unenhanced HRCT scan showed ongoing lung infiltrates with resolved chest effusions (Figure 3C), with the ongoing changes likely representing residual inflammation and cellular debris. The hsCRP decreased to 57 mg/L after 3 days of IVC treatment and normalized (<5 mg/L) after the 7-day IVC intervention (Figure 4A) (Table 2). Procalcitonin decreased to normal after 3 days of IVC treatment (0.35 mg/L) (Figure 4B) (Table 2). However, there was no significant improvement in coagulopathy by D-dimer, fibrinogen, and prothrombin time, which remained mildly elevated (Table 2). The patient was released from COVID-19 ICU on Hospital Day 18 and transferred to a community hospital for further quarantine and leukemia treatment. A follow-up unenhanced HRCT scan 31 days post-diagnosis showed largely resolved lung infiltrates and chest effusions (Figure 3D).

## 3. Discussion

Data from Wuhan, China, shows that COVID-19 results in a >20% mortality in cancer patients, several times higher than the reported 5–6% in the general affected population [18], and this cohort is at a higher risk because of their compromised immunity [19]. Oncology patients are also at higher risk for developing combined viral and bacterial infections and for worsened morbidity overall [4,18,19]. We discuss here two cases of COVID-19 in oncology patients with multiple severe comorbidities of cancer, including diabetes, anemia, and combined infections, in whom progression of disease was not controlled by general anti-viral, anti-bacterial, and anti-inflammatory treatments. The prognosis of both patients was poor, and neither was expected to survive. In fact, Patient 1 was moribund just prior to beginning intravenous vitamin C (IVC). In both patients, therapeutic intervention with IVC was begun at a total dose of 24 g/day given in divided doses for 7 days, with ongoing intensive supportive care. IVC seemed to effectively reduce systematic inflammation and improve patient outcomes. Both patients cleared SARS-CoV-2 after the IVC treatment and were released from the hospital. 

Notably, in both patients, with multiple failed anti-viral treatments and other interventions, including COVID-19 survivor serum, the addition of a 7-day course of IVC resulted in a negative SARS-CoV-2 test and overall symptomatic improvement. The improvements in clinical symptoms correlated in time with the IVC treatments. IVC treatment also reduced systematic inflammation as indicated by reduced hsCRP, IL-6, and procalcitonin levels. This suggests the benefit of using IVC as management for severe COVID-19 as demonstrated in both patients who otherwise were not expected to survive. 

The intervention of IVC began rather late in the course of the disease for these patients and was only started after patients failed other treatments, was critically ill, and not expected to survive. It appears that IVC unexpectedly enhanced viral clearance and reduced inflammation. It is reasonable to expect better effects if the IVC intervention is given earlier in the course of this viral illness. Of interest, patient two received oral 1.5 g of vitamin C daily from the outset of treatment, but there seemed to be no therapeutic benefit of oral dosing. It was only after intravenous infusions began that benefits were seen. This is believed to be related to IVC’s mechanism of action.

The mechanism (s) by which IVC exerts beneficial effects in COVID-19 is proposed from the authors’ prior work in cancer but remains to be fully elucidated. In cancer treatment, high dose IVC produces hydrogen peroxide (H_2_O_2_) at tumor tissue sites and thus induces death in cancer cells [10,20,21,22]. In the scenario of COVID-19, it is possible that H_2_O_2_ had direct viral deactivating effects. In addition, H2O2 and/or vitamin C may act as signaling transduction molecules and subsequently activate lymphocytes through epigenetic modulation [23,24,25,26]. Further, it is reported that in septic and ARDS patients, vitamin C is depleted [12], and the high IV dose may reverse depletion. Vitamin C as an antioxidant may reduce the damage caused by the “cytokine storm” often seen in COVID-19 patients and protect the endothelium integrity [27,28,29]. All these putative mechanisms are worth further investigation in COVID-19 and may lead to elucidating other mechanisms of action yet defined. 

Although there is a theoretic safety concern for H_2_O_2_ formation in the scenario of acute viral lung injury, our use of 12-g infusion given within 30–60 min did not exhibit any adverse effects. Extraordinary safety and benefit of IVC has also been reported by a group in Shanghai where doses equivalent to 10–20 g/day were used in more than 50 COVID-19 patients, and a 50 g infusion was used in a single COVID-19 patient [30]. In our two cases, vitamin C did not show therapeutic response until it was administered intravenously, as the oral supplementation of vitamin C at 1.5 g daily did not result in any benefit. As demonstrated in previous research, oral dosing is not expected to create plasma concentrations in a pharmacological range to produce effect [21,31]. Only intravenous dosing can bypass tight gut control for absorption and deliver pharmacologic doses that produce H_2_O_2_ formation.

It is important to note that both patients received standard anti-viral and anti-bacterial drugs, TCM treatments, and other anti-inflammatory drugs prior to and in conjunction with IVC, but the disease progressed before the IVC intervention. In addition, the possibility must be considered that IVC may have additive effects with the other supportive therapies in the treatment of COVID-19, as supportive care for symptoms is important to improve the patients’ clinical outcomes. We recognize the limitations of case studies and do not intend to generalize IVC efficacy in COVID-19 patients based on only these two cases. Rather, our observations are consistent with a case series from Shanghai in 50 COVID-19 patients treated with IVC, where benefits were observed [30]. 

Our case report suggests an unexpected benefit of using IVC as management for severe COVID-19 patients. More rigorous clinical studies should be conducted investigating the use of high dose intravenous vitamin C in COVID-19, with rationale based on mechanisms.

## Figures and Tables

**Table 1:** Patient 1 Laboratory Measurements and various markers over the course of hospitalization.

	Reference Range	IVC Treatment							
		Day 1	Day 8	Day 13	Day 15	Day 18	Day 20	Day 22	Day 24
***Pulse***	60–100 BPM	81	114	127	99	89	99	101	
***Respiratory rate***	12–20 BPM	15	28–43	31	28	25	32	22	
***Blood pressure***	90/60–120/80 mmHg	86/54	117/62	115/59	144/67	105/56	127/76	117/63	
***pO_2_ arterial***	80–100 mmHg	65		192.2	80.7	71	91.2	82.3	
***pCO_2_ arterial***	35–45 mmHg	37		120.7	72	78.4	67.6	52.2	
***pH arterial***	7.35–7.45	7.48		7.21	7.44	7.41	7.42	7.46	
***WBC***	3.5–9.5 G/L			16.39		7.56	5.59	6.04	
***Lymphocyte***	1.1–3.2 G/L	0.44	0.13	0.37	0.34	0.25	0.27	0.46	
***Neutrophil***	1.8–7.5 G/L	5.04	16.23	8.27	5.1	3.42	5.18	5.33	
***MPV***	8.2–12.5 fL	8	8				9.7	11.2	11.7
***Creatinine***	44–120 umol/L	42.1	28.9	39.3	55.8	56.9		41	
***Phosphors***	0.85–1.51 mmol/L	0.73			0.82	0.95	0.87		
***hsCRP***	0–5 mg/L	198	100	150		144	127	61	38
***Amyloid A***	0–8 mg/L	512		447					25
***IL-6***	0–16.4 pg/mL					94	36		22
***Prealbumin determination***	170–420 mg/L	16	93	10	16		41	58	102
***ALT***	0–50 U/L			55	8	12	9		
***AST***	0–40 U/L			150	13	31	27		
***BNP***	<100 ng/L	362		1411	765		1114	1475	1539
***Prothrombin time***	9–13 s	16		14.4	14.1	14.3	12.7	14	14
***PT ratio***	0.85–1.15	1.47		1.32	1.29	1.31	1.17	1.28	
***Fibrinogen***	2–4 g/L	5.55		4.98	1.72	5.01	4.62	3.68	3.67
***D-dimer***	0–0.25 mg/L	0.74		0.84	1.1	0.94	0.94	2.46	
***Fibrinogen degradation products***	0–5 mg/L	5.13				4.19	3.03	5.61	

**Table 2:** Patient 2. Laboratory measurements and various markers over the course of hospitalization.

	Reference Range	IVC Treatment					
		Day 2	Day 9	Day 10	Day 13	Day 16	Day 17
***Pulse***	60–100 BPM	88	74	70	81	72	89
***Respiratory rate***	12–20 BPM	17	21	18	17	19	20
***Blood pressure***	90/60–120/80 mmHg	110/59	124/70	119/69	124/64	118/72	123/43
***pO_2_ arterial***	80–100 mmHg		74.5		79.5		
***pCO_2_ arterial***	35–45 mmHg		31.6		29.3		
***HCO_3_ artierial***	22–26 mmol/L		19.3		22.9		
***Platelet***	125–350 G/L	79	105	115	129	138	
***Bilirubin***	<5.1 µmol/L	11.2	21.4	15.6	4.9		
***Glucose***	3.9–6.1 mmol/L	8.01	6.49		8.58		
***Creatinine***	44–120 umol/L	71.8	64.7		53.1		
***Phosphors***	0.85–1.51 mmol/L	1.08	1.07		1		
***hsCRP***	0–5 mg/L	36.25	63		57.43		<5
***Procalcitonin***	0–0.5 ng/mL			0.54	0.35		
***Prealbumin determination***	170–420 mg/L		134.7		84.7		
***Prothrombin time***	9–13 s	14.6	15.5	17.6	16.5		
***PT ratio***	0.85–1.15	1.34	1.42	1.61	1.51		
***Fibrinogen***	2–4 g/L	5.45	4.99	7.95	4.87		
***D-dimer***	0–0.25 mg/L	0.63	0.86	1.38	1.11		
***Fibrinogen degradation products***	0–5 mg/L			6.12	5.49		
***INR***	<1.5			1.59	1.5		
